# Variation in *CHI3LI* in Relation to Type 2 Diabetes and Related Quantitative Traits

**DOI:** 10.1371/journal.pone.0005469

**Published:** 2009-05-07

**Authors:** Camilla Noelle Rathcke, Johan Holmkvist, Torben Jørgensen, Knut Borch-Johnsen, Torben Hansen, Oluf Borbye Pedersen, Henrik Vestergaard

**Affiliations:** 1 Department of Endocrinology, Herlev Hospital, University of Copenhagen, Copenhagen, Denmark; 2 Hagedorn Research Institute, Gentofte, Denmark; 3 Research Centre for Prevention and Health, Glostrup Hospital, University of Copenhagen, Copenhagen, Denmark; 4 Steno Diabetes Center, Gentofte, Denmark; 5 Faculty of Health Sciences, University of Aarhus, Aarhus, Denmark; 6 Faculty of Health Sciences, University of Copenhagen, Copenhagen, Denmark; Mayo Clinic College of Medicine, United States of America

## Abstract

**Background:**

*CHI3LI* encoding the inflammatory glycoprotein YKL-40 is located on chromosome 1q32.1. YKL-40 is involved in inflammatory processes and patients with Type 2 Diabetes (T2D) have elevated circulating YKL-40 levels which correlate with their level of insulin resistance. Interestingly, it has been reported that rs10399931 (−329 G/A) of *CHI3LI* contributes to the inter-individual plasma YKL-40 levels in patients with sarcoidosis, and that rs4950928 (−131 C/G) is a susceptibility polymorphism for asthma and a decline in lung function. We hypothesized that single nucleotide polymorphisms (SNPs) or haplotypes thereof the *CHI3LI* locus might influence risk of T2D. The aim of the present study was to investigate the putative association between SNPs and haplotype blocks of *CHI3LI* and T2D and T2D related quantitative traits.

**Methods/Principal Findings:**

Eleven SNPs of *CHI3LI* were genotyped in 6514 individuals from the Inter99 cohort and 2924 individuals from the outpatient clinic at Steno Diabetes Center. In cas-control studies a total of 2345 T2D patients and 5302 individuals with a normal glucose tolerance test were examined.

We found no association between rs10399931 (OR, 0.98 (CI, 0.88–1.10), p = 0.76), rs4950928 (0.98 (0.87–1.10), p = 0.68) or any of the other SNPs with T2D. Similarly, we found no significant association between any of the 11 tgSNPs and T2D related quantitative traits, all p>0.14. None of the identified haplotype blocks of *CHI3LI* showed any association with T2D, all p>0.16.

**Conclusions/Significance:**

None of the examined SNPs or haplotype blocks of *CHI3LI* showed any association with T2D or T2D related quantitative traits. Estimates of insulin resistance and dysregulated glucose homeostasis in T2D do not seem to be accounted for by the examined variations of *CHI3LI*.

## Introduction

The primary molecular defects in Type 2 diabetes (T2D) remain largely unknown, but it is widely recognized that both genetic and environmental risk factors have interacting roles in the pathogenesis and the progression of the disease. However, recent discoveries have led to a progress in the understanding of the molecular genetic background of T2D brought forward by genome-wide association (GWA) studies which have dramatically increased the number of validated type 2 diabetes loci with modest impact on relative diabetes risk [1]–[3]. Several loci of interest may be located in the region of human chromosone 1q21–24 which shows replicated linkage to T2D in at least eight populations including North European Caucasian families, Pima Indians and Old Order Amish [1]. Furthermore, linkage analysis has also provided evidence for linkage of the metabolic syndrome (central obesity, dyslipidemia, hyperglycemia and hypertension) to 1q23–31 [4]. These regions as well as other closely related regions of chromosome 1 are under intense search for identification of T2D susceptibility genes.

Chitinase 3-like 1 (*CHI3LI*) encoding the inflammatory glycoprotein YKL-40 is located in a phylogenetically highly conserved area on chromosome 1q32.1. YKL-40 is involved in inflammatory processes and extracellular matrix remodelling including angiogenesis and functions as a growth factor for several cell types [5]. Studies have shown, that patients with T2D have elevated plasma YKL-40 levels [6], [7] which correlate with insulin resistance and with their diabetic lipid profile, but not with estimates of glycemic regulation [6]. Also patients with type 1 diabetes (T1D) have elevated YKL-40 levels which increase with increasing levels of albuminuria [8].

The *CHI3LI* promotor variant −329 G/A (rs10399931) associates with the inter-individual plasma YKL-40 levels in patients with sarcoidosis [9]. Furthermore, the functional *CHI3LI* promotor SNP −131C→G (rs4950928) increases the mRNA transcript levels coding for the YKL-40 protein in a genome wide study of gene expression in Epstein-Barr virus-transformed lymphoblastoid cells from children with asthma [10] and is shown to be predictive of asthma, bronchial hyperresponsiveness and reduced lung function [11]. Since *CHI3LI* is a susceptibility gene for asthma with elevated circulating YKL-40 levels as a biomarker, it is possible that one of these single nucleotide polymorphisms (SNPs) or another gene variation with which they could be in tight linkage disequilibrium with may be functional in relation to T2D.

Applying a HapMap–based tgSNP approach the objective of the present study was to investigate the putative association of common variation in the *CHI3LI* locus with T2D, obesity and several T2D associated quantitative traits.

## Methods

### Ethics Statement

Informed written consent was obtained from all participants before participation. The study was approved by the Ethical Committees of Copenhagen and Aarhus and was in accordance with the principle of the Helsinki Declaration II.

### Study population

A total of 11 tgSNPs (including the rs10399931 and rs4950928) covering all haplotype blocks in *CHI3LI* locus was genotyped. Genotyping was performed in 9438 individuals including Inter99, a population-based sample of unrelated, middle-aged Danish individuals living in the greater Copenhagen area, and 2924 individuals recruited from the outpatient clinic at Steno Diabetes Center.

The Inter99 study population has been described in detail previously [12]. In brief, both participants from the Inter99 cohort and individuals recruited from the outpatient clinic were characterized by an oral glucose tolerance test (OGTT). Impaired glucose homeostasis (IFG, IGT and diabetes) was defined according to WHO criteria after a 75 g OGTT [13] giving a classification of normal glucose tolerance (NGT; n = 5302), impaired fasting glycemia (IFG; n = 540), impaired glucose tolerance (IGT; n = 830) or screen-detected untreated T2D (SDU DM; n = 294). Missing data was seen in 421 individuals (4.5%) and a total of 2091 individuals had known T2D. The participants were investigated for an association between genotype and quantitative metabolic traits; individuals with known type 2 diabetes were excluded from these analyses. All participants were of Danish nationality.

### Biochemical and anthropometric measurements

Height and body weight were measured in light indoor clothes and without shoes, and BMI was calculated as weight (kg) divided by (height (m))^2^. Waist circumference was measured in the standing position midway between the iliac crest and the lower costal margin and hip circumference at its maximum.

In both Inter99- and Steno Diabetes Center participants, blood samples were drawn in the fasting state after a 12-h overnight fast. Plasma glucose was analysed by a glucose oxidase method (Granutest, Merck, Darmstadt, Germany). HbA_1C_ was measured by ion-exchange high performance liquid chromatography (normal reference range: 4.1–6.4%) and serum insulin (excluding des(31, 32) and intact proinsulin) was measured using the AutoDELFIA insulin kit (Perkin-Elmer/Wallac, Turku, Finland). Serum C-peptide concentrations were measured by a time-resolved fluoroimmunoassay (AutoDELFIA C-peptide kit; Perkin-Elmer/Wallac, Turku, Finland).

Serum triglyceride and total and HDL-cholesterol were analysed using enzymatic colorimetric methods (GPO-PAP and CHOD-PAP, Roche Molecular Biochemicals, Mannheim, Germany). HOMA-IR was calculated as fasting plasma glucose [mmol/l] multiplied by fasting serum insulin [pmol/l] and divided by 22.5. Insulinogenic index at 30 min was calculated as fasting serum insulin subtracted from plasma insulin at 30 min [pmol/l] divided by plasma glucose at 30 min [mmol/l].

### Genotyping

**Figure 1:**
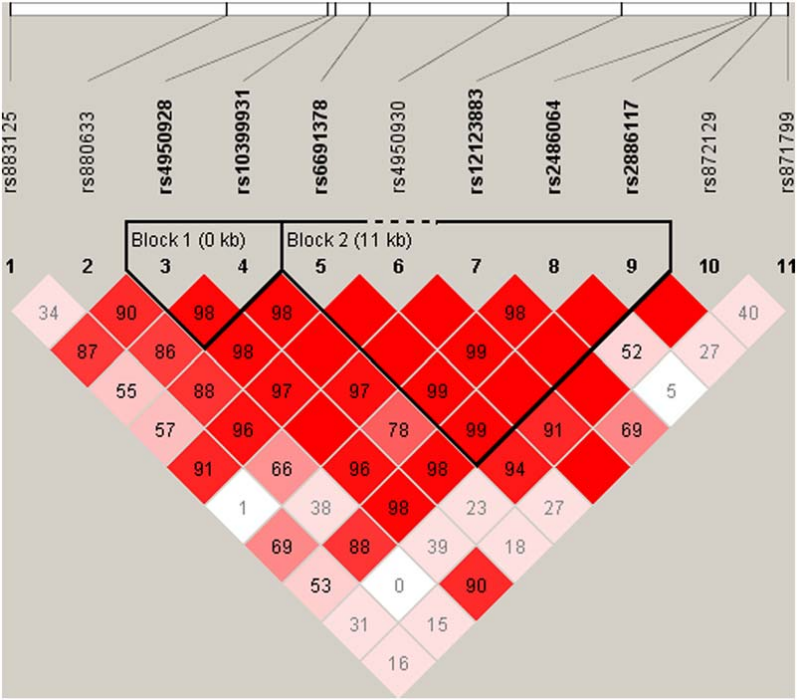
Linkage disequilibrium (LD) pattern of the investigated variations of *CHI3L1*.

SNPs in a region 22 kb upstream and 10 kb downstream of *CHI3LI* were chosen from the HapMap project (www.hapmap.org). Haplotype tgSNPs were selected from the HapMap data using TAGGER [14]. TAGGER was used with a 5% MAF cut off, agressive tagging (r^2^>0.8). Two haplotype blocks with 2 and 5 SNPs, respectively, and with similar structures in both patients with T2D and in controls were identified (Figure 1). The other tgSNPs were not included in the haplotype block analyses since they were not part of any block. Genotyping was performed using TaqMan allelic discrimination (Kbioscience, Herts, UK) with a success rate >97.0%. Discordance was 0% as judged from re-genotyping of 965 random duplicate samples. Genotype distribution obeyed Hardy Weinberg Equilibrium (HWE), all p>0.25.

### Statistical analyses

Logistic regression analyses were used to analyse differences in allele and genotype frequencies between case and control subjects; analyses were adjusted for age, sex and BMI where appropriate. The statistical analyses were performed using RGui version 2.5.0 (available at http://www.r-project.org). The haplotype block analyses were analysed for an association with T2D after adjustment for age, sex and BMI. Haplotype analyses were performed using PLINK [15]. P-values are not adjusted for multiple testing.

## Results

Clinical characteristics for the Inter99 cohort and the individuals recruited at Steno Diabetes Center are presented in Table 1. A total of 2345 T2D patients and 5302 individuals with a normal glucose tolerance test were identified.

**Table 1:** Clinical data for study sample.

	Complete sample	SDC sample	Inter99 cohort
N (m/w)	9438 (4834/4604)	2924 (1665/1259)	6514 (3169/3345)
NGT	5302 (2456/2846)	734 (334/400)	4568 (2122/2446)
IFG	540 (391/149)	32 (18/14)	508 (373/135)
IGT	830 (414/416)	123 (66/57)	707 (348/359)
SDU DM	294 (187/107)	39 (27/12)	255 (160/95)
Known DM	2051 (1259/801)	1946 (1200/755)	105 (59/46)
NGT age (years)	47±9	58±9	45±8
NGT BMI (kg/m^2^)	25.6±9.1	25.9±8.7	25.5±4.1
T2D age (years)	60±12	62±11	51±7
T2D BMI (kg/m^2^)	30.1±5.5	30.0±5.5	30.4±5.6
T2D HbA1c (%)	7.8±1.7	8.1±1.6	7.1±1.7

Data is mean±S.D. SDC, Steno Diabetes Center; N (m/w), number (men/women); NGT, normal glucose tolerance; IFG, impaired fasting glucose; IGT, impaired glucose tolerance; SDU DM, screen detected untreated diabetes mellitus; T2D, Type 2 diabetes; BMI, Body Mass Index; HbA1c, hemoglobin A1c.

Overall we found no association of rs10399931, rs4950928 or any of the other tgSNPs of *CHI3LI* with T2D after adjustment for age, sex, BMI and glucose tolerance, all p>0.25 (Table 2). In similar analyses, none of the tgSNPs were associated with impaired fasting glucose (IFG) or impaired glucose tolerance (IGT), although a borderline protective association with IGT was detected for rs12123883 (Table S1 and S2).

**Table 2:** Case control association studies of 2345 T2D patients and 5302 glucose tolerant control participants in relation to the 11 tgSNPs of *CHI3LI*.

SNP	Allele	MAF	Genotype	Genotype distribution		Additive model	p value
	(major/minor)	(%)	All	NGT, n (%)	T2D, n (%)	OR (CI)	
rs883125	C/G	15.6	CC	3546 (71.8)	1574 (71.0)	0.98 (0.86–1.12)	0.78
			CG	1280 (25.9)	591 (26.7)		
			GG	114 (2.3)	51 (2.3)		
rs880633	C/T	46.3	CC	1470 (30.0)	645 (29.4)	1.00 (0.90–1.10)	0.96
			CT	2416 (49.3)	1077 (48.8)		
			TT	1018 (20.7)	483 (21.8)		
rs4950928	C/G	20.4	CC	3159 (63.6)	1402 (63.4)	0.98 (0.87–1.10)	0.68
			CG	1612 (32.4)	716 (32.4)		
			GG	197 (4.0)	93 (4.2)		
rs10399931	C/T	23.7	CC	2894 (58.8)	1287 (58.4)	0.98 (0.88–1.10)	0.76
			CT	1754 (35.6)	791 (35.9)		
			TT	273 (5.6)	126 (5.7)		
rs6691378	G/A	12.6	GG	3840 (77.7)	1690 (76.4)	1.08 (0.94–1.25)	0.29
			GA	1030 (20.9)	487 (22.0)		
			AA	69 (1.4)	34 (1.6)		
rs4950930	G/A	4.0	GG	4512 (91.6)	2053 (92.5)	0.96 (0.76–1.20)	0.70
			GA	399 (8.1)	157 (7.1)		
			AA	13 (0.3)	10 (0.5)		
rs12123883	T/C	7.4	TT	4235 (85.5)	1906 (85.7)	0.95 (0.79–1.14)	0.58
			TC	684 (13.8)	305 (13.7)		
			CC	37 (0.7)	13 (5.8)		
rs2486064	G/A	42.3	GG	1636 (33.0)	742 (33.7)	0.97 (0.87–1.07)	0.48
			GA	2406 (48.6)	1057 (48.0)		
			AA	909 (18.4)	402 (18.3)		
rs2886117	G/A	12.9	GG	3809 (76.6)	1679 (75.8)	1.04 (0.90–1.20)	0.59
			GA	1078 (21.7)	500 (22.6)		
			AA	84 (1.7)	36 (1.6)		
rs872129	A/G	7.2	AA	4183 (84.5)	1910 (86.1)	0.91 (0.76–1.09)	0.32
			AG	730 (14.8)	298 (13.4)		
			GG	35 (0.7)	11 (0.5)		
rs871799	G/C	9.2	GG	4028 (81.3)	1821 (82.3)	0.96 (0.81–1.13)	0.60
			GC	873 (17.6)	376 (17.0)		
			CC	54 (1.1)	16 (0.7)		

T2D, type 2 diabetes; SNP, single nucleotide polymorphism; MAF, minor allele frequency; NGT, normal glucose tolerance; OR (CI), odds ratio (confidence interval).

Table 3 and 4 show the multiadjusted association of rs10399931 and rs4950928 with BMI and other T2D related quantitative traits among NGT, IFG, IGT and screen-detected untreated T2D in Inter99 and Steno Diabetes Center collected individuals. We found a significant association between rs10399931 and the serum C-peptide concentration after 2 hours, p = 0.03, but in all we found no significant association between rs10399931 or rs4950928 and any of the T2D quantitative traits. Similarly, none of the other SNPs showed any associations (data not shown).

**Table 3:** Association of rs10399931 of *CHI3LI* and T2D related quantitative traits among NGT, IFG, IGT and screen-detected untreated T2D in Inter99 and Steno Diabetes Center collected individuals.

Genotype	CC	CT	TT	P_add_
Alleles	4782	2927	781	
Age (years)	46±8	46±8	46±8	
BMI (kg/m^2^)	26.2±4.5	26.2±4.6	25.9±4	0.75
Whratio	0.86±0.09	0.86±0.09	0.86±0.09	0.89
Waist (cm)	87±13	87±13	86±13	0.69
Height (cm)	172±9	172±9	172±9	0.47
Fasting serum triglyceride (mmol/l)	1.3±1.5	1.3±1.1	1.3±0.8	0.75
Fasting serum cholesterol (mmol/l)	5.6±1.1	5.6±1.1	5.6±1.1	0.61
Fasting serum HDL cholesterol (mmol/l)	1.4±0.4	1.4±0.4	1.4±0.4	0.44
Systolic bp (mmHg)	130±17	131±17	131±17	0.41
Diastolic bp (mmHg)	82±11	83±11	82±12	0.34
Fasting plasma insulin (pmol/l)	42±28	42±27	41±27	0.98
Plasma insulin 30 min (pmol/l)	293±191	288±175	285±169	0.94
Plasma insulin 120 min (pmol/l)	217±217	215±203	224±208	0.16
HOMA-IR	10.6±8.1	10.5±7.7	10.4±7.7	0.95
Insulinogenic Index	30±20	29±19	29±18	0.75
Fasting plasma glucose (mmol/l)	5.5±0.8	5.5±0.9	5.5±0.7	0.84
Plasma glucose 30 min (mmol/l)	8.7±1.9	8.7±1.9	8.7±1.8	0.44
Plasma glucose 120 min (mmol/l)	6.2±2.2	6.2±2.2	6.3±2.1	0.71
Fasting plasma C-peptide (pmol/l)	596±274	593±256	594±273	0.53
Plasma C-peptide 30 min(pmol/l)	2006±736	1994±678	1977±732	0.86
Plasma C-peptide 120 min (pmol/l)	2305±1041	2315±980	2359±987	0.03

Data presented as mean (SD). Analyses adjusted for age, gender, BMI, glucose tolerance (NGT, IFG, IGT and screen-detected untreated T2D).

T2D, type 2 diabetes; NGT, normal glucose tolerance; IFG, impaired fasting glucose; IGT, impaired glucose tolerance; add, additive; dom, dominant; res, recessive; bmi, body mass index; whratio, waist-to-hip ratio; hdl, high density lipoprotein; bp, blood pressure; HOMA-IR, insulin resistance according to HOMA model.

**Table 4:** Association of rs49509028 of *CHI3LI* and T2D related quantitative traits among NGT, IFG, IGT and screen-detected untreated T2D in Inter99 and Steno Diabetes Center collected individuals.

Genotype	CC	CG	GG	P_add_
Alleles	5172	2635	334	
Age (years)	46±8	46±8	46±8	
BMI (kg/m^2^)	26.2±4.5	26.1±4.6	25.8±3.8	0.14
Whratio	0.86±0.09	0.86±0.09	0.86±0.09	0.43
waist (cm)	87±13	86±13	86±12	0.56
Height (cm)	172±9	172±9	172±9	0.91
Fasting serum triglyceride (mmol/l)	1.3±1.4	1.3±1.1	1.3±0.9	0.85
Fasting serum cholesterol (mmol/l)	5.6±1.1	5.6±1.1	5.6±1.1	0.28
Fasting serum HDL cholesterol (mmol/l)	1.4±0.4	1.4±0.4	1.4±0.4	0.36
Systolic bp (mmHg)	130±17	131±17	130±17	0.53
Diastolic bp (mmHg)	82±11	83±11	82±11	0.72
Fasting plasma insulin (pmol/l)	42±28	41±27	41±25	0.35
Plasma insulin 30 min (pmol/l)	295±191	287±174	276±151	0.36
Plasma insulin 120 min (pmol/l)	218±215	215±216	217±191	0.54
HOMA-IR	10.6±7.9	10.4±7.8	10.1±6.8	0.47
Insulinogenic Index	30±20	29±19	28±17	0.41
Fasting plasma glucose (mmol/l)	5.5±0.8	5.5±0.9	5.5±0.7	0.54
Plasma glucose 30 min (mmol/l)	8.7±1.9	8.7±1.8	8.7±1.8	0.94
Plasma glucose 120 min (mmol/l)	6.2±2.1	6.2±2.2	6.3±2.2	0.52
Fasting plasma C-peptide (pmol/l)	597±276	586±253	591±267	0.85
Plasma C-peptide 30 min(pmol/l)	2014±738	1988±676	1945±709	0.54
Plasma C-peptide 120 min (pmol/l)	2314±1042	2301±982	2336±968	0.17

Data presented as mean (SD). Analyses adjusted for age, gender, BMI, glucose tolerance (NGT, IFG, IGT and screen-detected untreated T2D).

T2D, type 2 diabetes; NGT, normal glucose tolerance; IFG, impaired fasting glucose; IGT, impaired glucose tolerance; add, additive; dom, dominant; res, recessive; bmi, body mass index; whratio, waist-to-hip ratio; hdl, high density lipoprotein; bp, blood pressure; HOMA-IR, insulin resistance according to HOMA model.

The two identified haplotype blocks of *CHI3LI* did not show any association with T2D (Table 5).

**Table 5:** *CHI3LI* haplotype association studies in relation to T2D.

SNPs	Haplotype	Frequency (%), total sample	p value
rs4950928 | rs10399931	GT	0.1999	0.71
	CT	0.0345	0.98
	CC	0.76398	0.74
rs6691378 | rs4950930 | rs12123883 | rs2486064 | rs2886117	GGTGA	0.00667	0.16
	GATGG	0.04281	0.47
	GGCAG	0.07954	0.54
	GGTAG	0.35018	0.65
	AGTGA	0.11792	0.46
	GGTGG	0.40448	0.38

Data show the association with T2D for the 2 haplotype blocks of 2 respectively 5 genotyped SNPs in *CHI3LI*. Haplotype tgSNPs were selected from the HapMap data using TAGGER.

T2D, type 2 diabetes; SNP, single nucleotide polymorphism.

## Discussion

This is a large-scale and to our knowledge the first study of variation in *CHI3LI* in relation to T2D and pre-diabetic quantitative traits. *CHI3LI* encodes the inflammatory protein YKL-40 which previously has been found to be elevated in plasma from patients with T2D [6], [7] and from patients with T1D where it also correlates with increasing levels of albuminuria [8]. In our study, we have exmained 11 tgSNPs covering the common variation (MAF>5%) in *CHI3LI* including the SNPs rs10399931 and rs4950928 which previously have been found to contribute to interindividual variations in circulating YKL-40 levels in patients with sarcoidosis [9] and asthma [11], respectively. Furthermore, *CHI3LI* has been found to be a susceptibility gene for asthma, since rs4950928 is predictive of asthma, bronchial hyperresponsiveness and reduced lung function.

A total of 9438 individuals have been examined, but no significant association of rs10399931, rs4950928 or any of the other tgSNPs of *CHI3LI* were found with T2D or any of the intermediate T2D related quantitative traits. A borderline protective association with IGT was detected for rs12123883, but rs12123883 is a rare SNP with only few homozygotes and the protective association is most likely a chance finding. Furthermore, the p-value would not withstand correction for multiple testing considering the number of tests performed. On the same line, none of the two identified haplotype blocks showed any association with T2D.

In association analyses of rs10399931 and SNPs analysed in the DGI genome wide association scan study, rs10399931 is in LD with rs4950929 (r^2^ = 0.74) and rs946263 (r^2^ = 0.78). Other correlations had r^2^-values<0.05. In the DGI genome wide association scan study, no significant associations of rs4950929 with any of the type 2 diabetic phenotypic traits were found, but the rs946263 G-allele was nominally found to be associated with T2D (p = 0.027) [16]. rs946263, which is a synonymous SNP located −9639 base pairs from the translational start site in *CHI3LI*, is also in perfect LD (r^2^ = 1.0) with rs4950928, which accounts for approximately 10% of the variance in plasma YKL-40 levels in asthma patients [11]. Beside the LD of rs10399931 and rs4950928 with rs946263, none of the examined SNPs or haplotype blocks of *CHI3LI* seem to be of any significance for the T2D diagnosis or any of the T2D related quantitative traits. Since the encoded inflammatory protein, YKL-40, of *CHI3LI* seems to be both a marker of inflammation and endothelial dysfunction [5], [17] it is possible that it is the endothelial dysfunction and the later micro- and macrovascular complications of T2D that accounts for the elevated YKL-40 levels in T2D patients. In endothelial dysfunction, elevated YKL-40 levels seem to play a role in relation to cell migration, reorganization and tissue remodelling as a response to endothelial damage [18]–[20].

The possible assocation between YKL-40 and vascular damage is supported by the finding of an independent association between elevated circulating YKL-40 levels and increasing levels of albuminuria in T1D patients [8]. Elevated plasma YKL-40 is also found to be associated with the presence and extent of coronary artery disease as assessed by coronary angiography [21] and just recently, YKL-40 levels have been found to be elevated in patients with myocardial infarction [22]. Furthermore, increasing YKL-40 levels do predict cardiovascular mortality in individuals without known DM or CHD after adjustment for known CV risk factors and markers [7].

Substantial evidence supports the notion that endothelial dysfunction and low-grade inflammation play a major pathophysiological role in the development of insulin resistance and the progression to manifest T2D [23]–[29]. We have previously shown, that YKL-40 levels are elevated in patients with T2D where it correlates with insulin resistance as assessed by the HOMA model and with levels of triglycerides and non-esterified fatty acids [6]. Since we found no association of any of the tgSNPs and surrogate markers of insulin resistance or the diabetic lipid profile in the present study, it does not seem to be any of these tgSNPs that explain the previously found correlation with these variables [6].

Multiple genes on different chromosomes may influence inflammatory biomarker levels and may have a potential role in the development of insulin resistance through a low-grade inflammatory state [30]–[33]. In proximity of the YKL-40 encoding gene, locus 1q32.2 has been documented to influence the plasma levels of ICAM-1[30], an adhesion molecule well-documented to participate in endothelial dysfunction and the development of T2D [23], [25]. However, association studies between YKL-40 and ICAM-1, either at protein or transcript levels, have not been conducted. Neither have association studies of variations of *CHI3L1* in relation to micro- or macrovascular conditions never been made.

In conclusion, both rs10399931 and rs4950928 are in high LD with rs946263 which is reported to associate with phenotypic traits of T2D in DGI [16]. However, neither rs1039991 nor rs4950928 nor any other tgSNPs of *CHI3LI* which were investigated in the present study are associated with T2D or T2D related quantitative traits including estimates of insulin resistance and dysregulated glucose homeostasis in the examined approximately 9500 Danes. Further studies are required to determine YKL-40 levels as a marker of inflammation in the prediabetic state and the role of *CHI3LI* in the pathogenesis of T2D.

## Supporting Information

Table S1 Case control association studies of 540 individuals with impaired fasting glucose (IFG) and 5302 glucose tolerant control participants in relation to the 11 tgSNPs of CHI3LI.
(0.08 MB DOC)

Table S2 Case control association studies of 830 individuals with impaired glucose tolerance (IGT) and 5302 glucose tolerant control participants in relation to the 11 tgSNPs of CHI3LI
(0.08 MB DOC)
