# Supplementary material for: Variation in CHI3LI in Relation to Type 2 Diabetes and Related Quantitative Traits
Source: PLoS One. 2009 May 7;4(5):e5469. doi: 10.1371/journal.pone.0005469 (PMC2674946; doi:10.1371/journal.pone.0005469)
Supplement: Table S1 — Case control association studies of 540 individuals with impaired fasting glucose (IFG) and 5302 glucose tolerant control participants in relation to the 11 tgSNPs of CHI3LI. (0.08 MB DOC) [file pone.0005469.s001.doc]

Table S1. Case control association studies of 540 individuals with impaired fasting glucose (IFG) and 5302 glucose tolerant control participants in relation to the 11 tgSNPs of *CHI3LI*.

| **SNP** | **Allele** | **MAF** | **Genotype** | **Genotype distribution** | | **Additive model** | **p value** |
| --- | --- | --- | --- | --- | --- | --- | --- |
|  | (major/minor) | (%) | All | **NGT, n (%)** | **IFG, n (%)** | **OR (CI)** |  |
| rs883125 | C/G | 15.6 | CC | 3536 (71.8) | 336 (69.0) | 1.09 (0.91-1.31) | 0.36 |
|  |  |  | CG | 1278 (25.9) | 139 (28.5) |  |  |
|  |  |  | GG | 113 (2.3) | 12 (2.5) |  |  |
| rs880633 | C/T | 46.3 | CC | 1463 (29.9) | 150 (30.9) | 1.00 (0.87-1.14) | 0.98 |
|  |  |  | CT | 2409 (49.3) | 234 (48.1) |  |  |
|  |  |  | TT | 1017 (20.8) | 102 (21.0) |  |  |
| rs4950928 | C/G | 20.4 | CC | 3148 (63.5) | 324 (65.6) | 0.95 (0.80-1.13) | 0.58 |
|  |  |  | CG | 1608 (32.5) | 150 (30.4) |  |  |
|  |  |  | GG | 197 (4.0) | 20 (4.0) |  |  |
| rs10399931 | C/T | 23.7 | CC | 2884 (58.8) | 305 (60.0) | 0.95 (0.81-1.12) | 0.54 |
|  |  |  | CT | 1749 (35.7) | 178 (35.1) |  |  |
|  |  |  | TT | 273 (5.5) | 25 (4.9) |  |  |
| rs6691378 | G/A | 12.6 | GG | 3829 (77.7) | 381 (78.4) | 0.92 (0.74-1.14) | 0.42 |
|  |  |  | GA | 1027 (20.9) | 102 (21.0) |  |  |
|  |  |  | AA | 69 (1.4) | 3 (0.6) |  |  |
| rs4950930 | G/A | 4.0 | GG | 4500 (91.6) | 445 (90.8) | 1.04 (0.75-1.44) | 0.81 |
|  |  |  | GA | 398 (8.1) | 45 (9.2) |  |  |
|  |  |  | AA | 13 (0.3) | 0 (0.0) |  |  |
| rs12123883 | T/C | 7.4 | TT | 4223 (85.5) | 423 (86.0) | 0.93 (0.72-1.21) | 0.60 |
|  |  |  | TC | 682 (13.8) | 67 (13.6) |  |  |
|  |  |  | CC | 37 (0.7) | 2 (0.4) |  |  |
| rs2486064 | G/A | 42.3 | GG | 1631 (33.0) | 172 (35.0) | 0.90 (0.78-1.03) | 0.13 |
|  |  |  | GA | 2397 (48.6) | 250 (50.8) |  |  |
|  |  |  | AA | 908 (18.4) | 70 (14.2) |  |  |
| rs2886117 | G/A | 12.9 | GG | 3798 (76.6) | 379 (77.2) | 0.93 (0.76-1.15) | 0.51 |
|  |  |  | GA | 1075 (21.7) | 107 (21.8) |  |  |
|  |  |  | AA | 84 (1.7) | 5 (1.0) |  |  |
| rs872129 | A/G | 7.2 | AA | 4183 (84.5) | 412 (84.3) | 1.01 (0.79-1.30) | 0.93 |
|  |  |  | AG | 730 (14.8) | 75 (15.3) |  |  |
|  |  |  | GG | 35 (0.7) | 2 (0.4) |  |  |
| rs871799 | G/C | 9.2 | GG | 4028 (81.3) | 398 (80.9) | 1.05 (0.85-1.31) | 0.64 |
|  |  |  | GC | 873 (17.6) | 87 (17.7) |  |  |
|  |  |  | CC | 54 (1.1) | 7 (1.4) |  |  |

SNP, single nucleotide polymorphism; MAF, minor allele frequency; NGT, normal glucose tolerance; OR (CI), odds ratio (confidence interval).
