# Supplementary material for: Variation in CHI3LI in Relation to Type 2 Diabetes and Related Quantitative Traits
Source: PLoS One. 2009 May 7;4(5):e5469. doi: 10.1371/journal.pone.0005469 (PMC2674946; doi:10.1371/journal.pone.0005469)
Supplement: Table S2 — Case control association studies of 830 individuals with impaired glucose tolerance (IGT) and 5302 glucose tolerant control participants in relation to the 11 tgSNPs of CHI3LI (0.08 MB DOC) [file pone.0005469.s002.doc]

Table S2. Case control association studies of 830 individuals with impaired glucose tolerance (IGT) and 5302 glucose tolerant control participants in relation to the 11 tgSNPs of *CHI3LI*.

| **SNP** | **Allele** | **MAF** | **Genotype** | **Genotype distribution** | | **Additive model** | **p value** |
| --- | --- | --- | --- | --- | --- | --- | --- |
|  | (major/minor) | (%) | All | **NGT, n (%)** | **IGT, n (%)** | **OR (CI)** |  |
| rs883125 | C/G | 15.6 | CC | 3536 (71.8) | 346 (71.3) | 1.03 (0.86-1.24) | 0.73 |
|  |  |  | CG | 1278 (25.9) | 125 (25.8) |  |  |
|  |  |  | GG | 113 (2.3) | 14 (2.9) |  |  |
| rs880633 | C/T | 46.3 | CC | 1463 (29.9) | 136 (28.1) | 1.07 (0.94-1.23) | 0.29 |
|  |  |  | CT | 2409 (49.3) | 239 (49.4) |  |  |
|  |  |  | TT | 1017 (20.8) | 109 (22.5) |  |  |
| rs4950928 | C/G | 20.4 | CC | 3148 (63.5) | 287 (60.1) | 1.15 (0.98-1.36) | 0.08 |
|  |  |  | CG | 1608 (32.5) | 167 (34.9) |  |  |
|  |  |  | GG | 197 (4.0) | 24 (5.0) |  |  |
| rs10399931 | C/T | 23.7 | CC | 2884 (58.8) | 296 (55.7) | 1.10 (0.95-1.27) | 0.22 |
|  |  |  | CT | 1749 (35.7) | 204 (38.3) |  |  |
|  |  |  | TT | 273 (5.5) | 32 (6.0) |  |  |
| rs6691378 | G/A | 12.6 | GG | 3829 (77.7) | 369 (75.5) | 1.13 (0.92-1.38) | 0.24 |
|  |  |  | GA | 1027 (20.9) | 113 (23.1) |  |  |
|  |  |  | AA | 69 (1.4) | 7 (1.4) |  |  |
| rs4950930 | G/A | 4.0 | GG | 4500 (91.6) | 451 (93.2) | 0.80 (0.56-1.14) | 0.22 |
|  |  |  | GA | 398 (8.1) | 33 (6.8) |  |  |
|  |  |  | AA | 13 (0.3) | 0 (0.0) |  |  |
| rs12123883 | T/C | 7.4 | TT | 4223 (85.5) | 440 (90.3) | 0.63 (0.46-0.85) | 0.0025 |
|  |  |  | TC | 682 (13.8) | 46 (9.5) |  |  |
|  |  |  | CC | 37 (0.7) | 1 (0.2) |  |  |
| rs2486064 | G/A | 42.3 | GG | 1631 (33.0) | 174 (35.9) | 0.93 (0.81-1.07) | 0.32 |
|  |  |  | GA | 2397 (48.6) | 224 (46.3) |  |  |
|  |  |  | AA | 908 (18.4) | 86 (17.8) |  |  |
| rs2886117 | G/A | 12.9 | GG | 3798 (76.6) | 360 (74.1) | 1.15 (0.95-1.40) | 0.14 |
|  |  |  | GA | 1075 (21.7) | 116 (23.9) |  |  |
|  |  |  | AA | 84 (1.7) | 10 (2.0) |  |  |
| rs872129 | A/G | 7.2 | AA | 4183 (84.5) | 423 (87.0) | 0.85 (0.66-1.10) | 0.22 |
|  |  |  | AG | 730 (14.8) | 57 (11.7) |  |  |
|  |  |  | GG | 35 (0.7) | 6 (1.3) |  |  |
| rs871799 | G/C | 9.2 | GG | 4028 (81.3) | 400 (82.3) | 0.95 (0.76-1.20) | 0.68 |
|  |  |  | GC | 873 (17.6) | 80 (16.5) |  |  |
|  |  |  | CC | 54 (1.1) | 6 (1.2) |  |  |

SNP, single nucleotide polymorphism; MAF, minor allele frequency; NGT, normal glucose tolerance; OR (CI), odds ratio (confidence interval).
